# *QuickStats:* Birth Rates* for Females Aged 15–19 Years, by State — National Vital Statistics System, United States, 2021

**DOI:** 10.15585/mmwr.mm7150a8

**Published:** 2022-12-16

**Figure:**
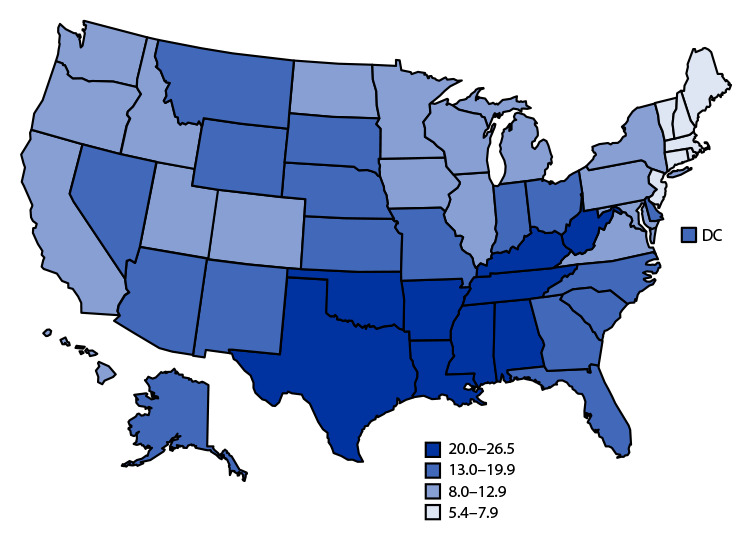
In 2021, the U.S. birth rate for females aged 15–19 years was 13.9 births per 1,000 persons, with rates generally lower in the Northeast and higher across the southern states. Birth rates among females aged 15–19 years ranged from 5.4 in New Hampshire, 5.7 in Massachusetts, and 6.4 in Vermont to 26.5 in Arkansas and 25.6 in Mississippi.

**Source:** National Center for Health Statistics, National Vital Statistics System, Natality Data, 2021. https://www.cdc.gov/nchs/nvss/births.htm

